# Exploration and comparison of methods for combining population- and family-based genetic association using the Genetic Analysis Workshop 17 mini-exome

**DOI:** 10.1186/1753-6561-5-S9-S28

**Published:** 2011-11-29

**Authors:** David W Fardo, Anthony R Druen, Jinze Liu, Lucia Mirea, Claire Infante-Rivard, Patrick Breheny

**Affiliations:** 1 Department of Biostatistics, University of Kentucky College of Public Health, 121 Washington Avenue, Lexington, KY 40536, USA; 2 Division of Biomedical Informatics, University of Kentucky College of Public Health, 121 Washington Avenue, Lexington, KY 40536, USA; 3 Center for Clinical and Translational Science, University of Kentucky, 800 Rose Street, Room C-300 , Lexington, KY 40536, USA; 4 Department of Computer Science, University of Kentucky, 329 Rose Street, Lexington, KY 40506, USA; 5 Dalla Lana School of Public Health, University of Toronto, 155 College Street, Health Science Building, 6th floor, Toronto, ON M5T 3M7, Canada; 6 Samuel Lunenfeld Research Institute Mount Sinai Hospital Joseph and Wolf Lebovic Health Complex, 600 University Avenue, Toronto, ON M5G 1X5, Canada; 7 Department of Epidemiology, Biostatistics and Occupational Health, McGill University, Montreal, Canada Purvis Hall, 1020 Pine Avenue West, Montreal, QC H3A 1A3, Canada

## Abstract

We examine the performance of various methods for combining family- and population-based genetic association data. Several approaches have been proposed for situations in which information is collected from both a subset of unrelated subjects and a subset of family members. Analyzing these samples separately is known to be inefficient, and it is important to determine the scenarios for which differing methods perform well. Others have investigated this question; however, no extensive simulations have been conducted, nor have these methods been applied to mini-exome-style data such as that provided by Genetic Analysis Workshop 17. We quantify the empirical power and false-positive rates for three existing methods applied to the Genetic Analysis Workshop 17 mini-exome data and compare relative performance. We use knowledge of the underlying data simulation model to make these assessments.

## Background

Study designs for genetic association studies fall into two broad categories: (1) population-based studies that recruit unrelated individuals and (2) family-based studies that collect some number of related pedigrees. Often, both study designs are used for a particular investigation. For example, when a linkage study has been performed and family data are collected, follow-up analysis can include association using a new unrelated study population. The analytic methods appropriate for either design differ, thus making difficult the aggregation of the association metrics across the study designs. Heuristically, population-based metrics attempt to quantify a measure of correlation or association between some function of genotype at a given marker and the disease phenotype, whereas family-based association measures use properties of Mendelian transmissions from parents to offspring and are inherently conditional.

Because analyzing the disparate types of data in isolation most often results in nonoptimal statistical power, investigators have proposed several methods for efficiently combining these data. We briefly summarize three methods to be applied to the Genetic Analysis Workshop 17 (GAW17) data in the Methods section. Each approach is distinguished by the study designs for which it is appropriate, the assumptions necessary for valid inference, and the handling of population stratification (whether it is formally or informally tested or whether it is taken into account by means of adjustments). Operationally, these methods are distinguishable by computation and implementation considerations and by empirical performance. We assess the performance in this paper. Other researchers have investigated the question of relative performance [1]; however, no simulations have been conducted for comparison.

An important consideration to keep in mind throughout this investigation is the underlying causal model that was used to generate the GAW17 data [2]. First, rather than reflecting the common disease/common variant hypothesis that the established methods presented address, the data-generating mechanism used was consistent with the multiple rare variant or the common disease/rare variant (CDRV) hypothesis, which suggests that common disease susceptibility is garnered through multiple rare variants with moderate to high penetrance. Intuitively, the current methods do not perform well in identifying rare single-nucleotide polymorphisms (SNPs); in this paper we intend to assess this performance and to motivate possible modifications that would be successful when the CDRV hypothesis is true. In addition, the disease was simulated to have ≫ 30% prevalence, which violates the often-invoked rare disease assumption.

## Methods

The first attempts to combine population- and family-based association data were developed by Nagelkerke et al. [3], who used a likelihood framework to combine case-control data with family data by exploiting the likelihood formulation [4] of the transmission disequilibrium test (TDT) [5]. This approach assumes Hardy-Weinberg equilibrium (HWE), random mating, and a multiplicative model of allelic effect. Although no formal test of the appropriateness of combining the two types of data has been developed, we discuss ad hoc procedures.

Epstein et al. [6] generalized this work by relaxing the assumptions of HWE, random mating, and the assumed multiplicative mode of inheritance. In addition, they described a formal test for the appropriateness of combining case-control and case-trio data by comparing genotype relative risk (RR) estimates from between-individual and within-family analyses, respectively. The proposed two-stage procedure facilitates valid model selection in the presence of population stratification. Further extensions of this approach were made by Chen and Lin [7]. Their method uses weighted least squares to aggregate the disparate RRs and requires no assumptions for mating-type distributions.

Epstein et al.’s and Chen and Lin’s methods rely on two strong assumptions: a rare disease and the absence of population stratification. Later work has been targeted at both relaxing the rare disease assumption and adjusting for population stratification. Zhu et al. [8] used a principal components strategy to adjust for population stratification and to aggregate families and case-control samples by means of a linear regression framework. Within-family correlations were empirically estimated from the data and incorporated into the variance of the test statistic. Zhang et al. [9] proposed a similar method in which they defined a score test and used generalized estimating equations [10] to account for familial correlation. Their method can be more easily applied to multivariate outcomes. Other useful approaches, some with a focus on genome-wide association, have been proposed but are not evaluated here [11-21].

Because the approach by Chen and Lin [7] is not immediately generalizable to pedigrees, we extracted nuclear families and then sampled 194 trios from the nuclear families to provide a uniform comparison between the methods. These sampled data (697 unrelated case or control individuals and 582 family members from the 194 trios) are used for our comparisons. We assume an additive mode of inheritance throughout.

### Chen and Lin’s method

Chen and Lin’s [7] approach uses the conditional on parental genotypes (CPG) approach of Schaid and Sommer [22] to construct the likelihood of the case-trio samples. An estimate for the RR is obtained from the CPG likelihood and is denoted . This estimate is then compared to a traditional logistic regression estimate of the genotype log odds ratio, , using the case-control sample, which is composed of case-trio probands and the unrelated control subjects. Chen and Lin use a Wald-type test to determine whether the effect estimates are consistent. If this test is not rejected, a weighted least-squares estimator for the combined genetic effect is then constructed for inference as:(1)

where *W*_1_ and *W*_2_ are weights derived from linear model theory assuming the parameter estimates follow a multivariate normal distribution (see Chen and Lin [7] for details). Here, the assumptions of a rare disease and no population stratification are necessary for validity. However, the test used to reject the appropriateness of combining the RR estimates is not well powered, as evidenced by our simulations, which often did not confer sufficient evidence to reject the null hypothesis of parameter equivalence even though the simulated disease is not, in fact, rare—a necessary condition for such equivalence. This method was designed for case-trio and unrelated control subjects; however, in our analyses control offspring from the control trios are added to the case-control subsample.

### Zhu et al.’s method

In Zhu et al.’s [8] approach, principal components are calculated from the genotypes of all unrelated individuals (trio parents and unrelated case and control subjects), and both the genotypes and the phenotypes of these individuals are then separately regressed on the principal components. The resulting linear regression parameter estimates are used to calculate genotypic and phenotypic residuals,  and , respectively, where *i* indexes families and *j* indexes individuals within a family. The covariance between these residuals is measured as:(2)

where *N* is the number of families, *k_i_* is the number of individuals in the *i*th family, and *N_T_* is the total number of individuals. Within-family correlations are taken into account in the calculation of the variance of *T* to construct a Wald test. Although this method requires enough markers to estimate principal components, it has the distinct advantage of being robust to population stratification. It can incorporate more complex family structures and does not discard any of the GAW17 data for analysis. Software to apply this approach, FamCC, is available from Zhu et al. [8].

### Zhang et al.’s method

Zhang et al.’s [9] method adapts a score test statistic proposed by Lange et al. [23] that applies generalized estimating equations to family-based association tests. To obtain estimates for the score test statistic, the components of the test statistic are decomposed into two mutually exclusive sets: the unrelated individuals and the trios. Traits are treated as constants so that the population genotype mean and variance are estimated for the unrelated individuals and the genotype mean and variance for the offspring are defined through Mendelian transmissions. Similar to Zhu et al.’s method, this framework allows for incorporation of covariates, but unlike the other methods considered, it can easily handle missing parents.

Zhang et al. [9] use principal components analysis (PCA) to adjust for population stratification. This is done separately for the two data subsets. The standard principal-components-based adjustment is used for the unrelated individuals in order to adjust the corresponding genotype and phenotype vectors by means of linear regression on the principal components, which results in:(3)

where  and  are the adjusted population trait and genotype means, respectively. A TDT-like PCA that adjusts for population stratification in family data [24] is used within the set of related individuals to define:(4)

where *g_im_* and *g_if_* are the mother’s and father’s genotypes in the *i*th family, respectively. The score *Z* = *U* + *R* is squared and standardized by its variance to provide a score test. Zhang et al. [9] provide a Java-based program, GAP, for analysis.

## Results

For each method we tested all 24,487 SNPs from the GAW17 data using the 697 unrelated individuals in the case-control sample and the subsampled 194 trios (582 individuals) in each of the 200 simulation replicates, with affected status as the phenotype. Although an adjustment for multiple testing would be appropriate for this study design, we chose to use a 5% nominal level of significance throughout in order to better compare the methods. Although these methods readily generalize to handling other genetic models, we assumed an additive mode of inheritance throughout.

### False-positive rates

Table 1 displays the average rejection rates across all noncausal and causal SNPs for each aggregation method. Although error rate inflation does not appear to be a problem, it is easy to see that all methods are low powered and that only the Zhang et al. [9] approach appears to have a discernible increase in the rejection rates from the null SNPs to the causal SNPs. It also appears that removing so-called spurious genes [25] from the noncausal SNPs lowers the error rate, as expected.

**Table 1:** Average empirical rejection rates

Method	Noncausal SNPs		Causal SNPs
	
	All	With SNPs from spurious genes removed	All
Chen and Lin	0.0388	0.0378	0.0269
Zhang et al.	0.0420	0.0408	0.0761
Zhu et al.	0.0551	0.0551	0.0556

Empirical rejection rates over the 200 replications for each method averaged over all SNPs (24,327 noncausal SNPs and 160 causal SNPs, 2 of which confer susceptibility through two different components of the latent disease susceptibility distribution). Removing SNPs from the spurious genes [25] results in 16,380 noncausal SNPs.

### SNP discovery power

**Figure 1:**
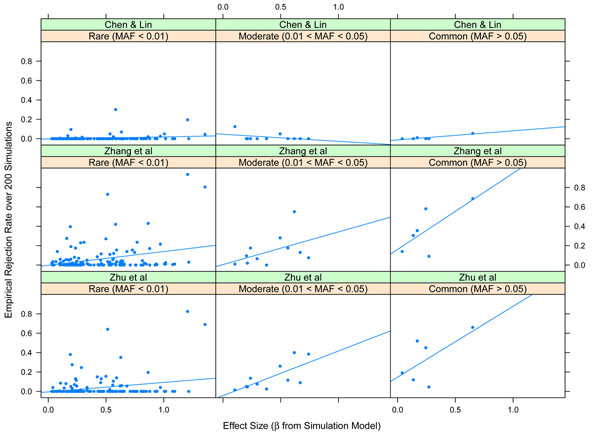
**Empirical rejection rates for causal SNPs.** Empirical rejection rate of each method to select causal SNPs. True effect size (*β*) is on the horizontal axis, and the strips correspond to rare, moderate, or common SNPs. MAF, minor allele frequency.

Although the power averaged over causal SNPs was low, some of the SNPs were detectable at high rates. Figure 1 displays the empirical powers for each method plotted against the effect size and grouped into three categories of SNP minor allele frequency. Here, effect size is not directly for disease status but rather for an underlying distribution of disease susceptibility [2]. It is clear that many rare SNPs are not detectable for any of the examined methods. However, contrary to intuition, many of the rarer SNPs provide the highest levels of power. Those SNPs with substantive power vary between small and large effect sizes. Examining SNPs for which there is at least modest power (Table 2) reveals that the Zhang et al. [9] approach most often is the highest powered.

**Table 2:** Empirical rejection rates for top causal SNPs

Causal SNP	Gene	Effect size	MAF	Chen and Lin	Zhang et al.	Zhu et al.
C1S3181	*ELAVL4*	0.30946	0.000717	0	**0.235**	0
C1S3181	*ELAVL4*	0.76911	0.000717	0	**0.235**	0
C1S9189	*PIK3C2B*	0.19102	0.006456	0	**0.395**	0.380
C3S4880	*BCHE*	0.20651	0.001435	0	0.005	**0.275**
C4S1873	*KDR*	0.58301	0.000717	0.300	**0.420**	0
C4S1878	*KDR*	0.13573	0.164993	0	**0.305**	0.120
C4S4935	*VEGFC*	1.35726	0.000717	0.045	**0.805**	0.690
C5S5133	*FLT4*	0.15986	0.001435	0	**0.275**	0
C6S2981	*VEGFA*	1.20645	0.002152	0.195	**0.935**	0.825
C6S5380	*VNN1*	0.24437	0.170732	0	**0.580**	0.450
C8S442	*LPL*	0.49459	0.015782	0.050	**0.280**	0.260
C9S444	*VLDLR*	0.86528	0.001435	0.020	**0.430**	0.195
C10S3050	*SIRT1*	0.97060	0.002152	0.025	**0.215**	0.040
C10S3109	*SIRT1*	0.51421	0.000717	0	**0.730**	0.640
C13S431	*FLT1*	0.74136	0.017217	0	0.075	**0.385**
C13S522	*FLT1*	0.61830	0.027977	0	**0.550**	0.400
C13S523	*FLT1*	0.64997	0.066714	0.055	**0.685**	0.660
C14S1382	*SOS2*	0.28058	0.003587	0	**0.230**	0.015
C17S1043	*SREBF1*	0.49941	0.004304	0	**0.270**	0.155
C17S1046	*SREBF1*	0.62779	0.002869	0	0.015	**0.350**
C17S1048	*SREBF1*	0.28739	0.001435	0	0	**0.245**
C17S4578	*PRKCA*	0.17038	0.166428	0.010	0.355	**0.520**

Gene, effect size, minor allele frequency (MAF), and empirical rejection rate over the 200 replications from each method for the 21 causal SNPs conferring ≥20% empirical rejection rate from at least one of the three methods. The maximum empirical rejection rate over the three methods is in boldface for each causal SNP. There are 160 causal SNPs, 2 of which confer susceptibility through two different components of the latent disease susceptibility distribution.

## Discussion and conclusions

Several methods address the problem of combining population- and family-based genetic association data. These methods differ fundamentally in whether they incorporate within-family transmissions and rely on tests for population stratification to justify effect estimate aggregation or perform between-individual analyses using family data. Performance related to population stratification cannot be assessed here because no stratification was simulated in the GAW17 data.

Although the Zhang et al. [9] method performed better than the other two methods considered, we did see that no method was well powered to detect causal SNPs in this scenario. Both the Zhang et al. [9] and the Zhu et al. [8] methods allow for more general pedigree structures than the trios-only analysis performed here and will likely perform more favorably when larger pedigrees are considered. In future work, we plan to adapt aggregation methods suitable for the CDRV hypothesis.

## Competing interests

The authors declare that there are no competing interests.

## Authors’ contributions

The study was conceived by DWF, LM and PB. DWF, ARD and PB ran the analyses. DWF and PB summarized the results and created figures. DWF and PB drafted the manuscript, which was revised by JL, LM and CIR. All authors read and approved the final manuscript.
